# The Complexity of Deep Acting: A Study of Emotional Labor in Frontline Human Service Work

**DOI:** 10.1177/21568693251345052

**Published:** 2025-06-30

**Authors:** Diana Singh, Ruth Repchuck, Jessica Monaghan

**Affiliations:** 1 McMaster University, Hamilton, ON, Canada

**Keywords:** emotions, stress exposure, work stress, mental health

## Abstract

This study examines the mental health impacts of emotional labor using an integrated analytical model that considers both job-level emotional labor requirements (negative and positive display rules) and psychological processes of emotion regulation (deep acting and surface acting). Using data from the 2021 Emotions Matter Survey (*n* = 854) of mental health and library workers in Ontario, Canada, we explore the conditions under which emotional labor requirements negatively impact mental health. Our findings reveal that negative display rules significantly increase anxiety, and surface acting exacerbates both anxiety and anger. Contrary to expectations, deep acting did not serve as a protective factor for mental health but showed a complex interaction with negative display rules, worsening psychological states under high negative emotional demands. This research underscores the intricate nature of emotional labor’s impact on mental health, highlighting the need for context-specific strategies to support frontline workers in emotionally intensive roles.

In postpandemic research, the concept of emotional labor has emerged as a focal point of examination within the realm of mental health, particularly for frontline service workers who often navigate complex emotional interactions as part of their daily work experiences (O’Neil et al. 2023; Salvesen and Berg 2021). Emotional labor, defined as the process of managing feelings and expressions to fulfill the emotional requirements of a job, encompasses a range of practices, from displaying positive emotions to suppressing negative ones, in accordance with organizational or occupational norms (Grandey, Diefendorff, and Rupp 2013). Emotional labor research is rooted in Hochschild’s (1983) seminal work, which highlighted the psychological costs of managing emotionsin service-oriented occupations (e.g., emotional exhaustion, dissonance, and burnout). Since this piece, the discourse has expanded to explore the nuanced dimensions of emotional labor, including the dichotomy of positive and negative display rules and the psychological strategies workers employ to regulate their emotions, namely, surface acting and deep acting (Grandey and Gabriel 2015; Grandey and Melloy 2017). However, despite the emergence of newer conceptual models, the exploration of emotional labor through an integrated process model, that closely associates with the internal psychological strategies of emotion regulation, remains an underexplored area of research.

The distinction between surface acting and deep acting as emotion regulation strategies underlines a critical area of investigation (Gabriel, Diefendorff, and Grandey 2023). While surface acting involves altering external expressions without changing internal feelings, deep acting seeks to align one’s internal emotional state with external displays, theoretically mitigating the potential negative effects of emotional dissonance (Ashforth and Humphrey 1993; Hoffmann 2016). Despite the intuitive appeal of deep acting as a healthier emotion regulation strategy, empirical findings have been mixed, suggesting a complex interplay between the type of emotional labor performed, the strategies used for emotion regulation, and their impacts on mental health (Grandey and Melloy 2017; Huppertz et al. 2020). This inconsistency in the literature underscores the need for a more granular investigation into how these elements interact within the high-stress environments that frontline workers often inhabit.

Methodological approaches in the study of emotional labor have varied widely, ranging from self-reported assessments of emotional labor experiences to more objective, occupation-level measures (Kruml and Geddes 2000; Wharton 2009). While individual-level studies provide insight into the personal perceptions and frequencies of emotional labor, occupational-level research offers a broader, aggregate view of emotional requirements across various jobs. This methodological diversity has enriched the discourse, allowing for a nuanced understanding of emotional labor’s impact across different work contexts (Grandey and Gabriel 2015); However, it may also have contributed to inconsistent findings with regards to the impact of emotion regulation as an intermediary influence on mental health.

Against this backdrop, our study addresses several research questions: (1) How do the requirements of emotional labor, categorized into negative and positive display rules, affect the mental health of frontline service workers? (2) What role do emotion regulation strategies, specifically surface and deep acting, play in moderating these effects? (3) Does the impact of these emotion regulation strategies vary depending on the type of display rules enforced?

To address these questions, we use data from the 2021 Emotions Matter Survey (*n* = 854), focusing on a sample of frontline workers in mental health and addictions, as well as library services, during the early stages of the COVID-19 pandemic in Ontario, Canada. This period, marked by heightened emotional demands and stressors, provides a unique lens through which to examine the implications of emotional labor requirements and emotion regulation on workers’ mental health. In addition, this study examines multiple mental health outcomes including, generalized anxiety, self-rated mental health and anger—both internalizing (e.g., anxiety) and externalizing (e.g., anger) in the same framework. While previous emotional labor research has predominantly focused on internalizing outcomes, this dual focus aligns with the stress process model, which emphasizes the importance of capturing diverse dimensions of mental health to understand the full impact of social stressors (Aneshensel 2005).

## Literature Review

### Emotional Labor

The concept of emotional labor initially stemmed from Arlie Hochschild’s influential research, *The Managed Heart (1983)*, where she demonstrated how it had been institutionalized within the airline industry. Hochschild (1983) used flight attendants as a compelling case study that drew attention to the potential mental health consequences for workers where managing emotions is a key aspect of their day-to-day work. Since then, emotional labor research has found that requirements of emotional labor, such as suppressing felt emotions, are associated with increased burnout, anxiety and emotional exhaustion (Chen et al. 2022; Clarke et al. 2021; O’Neil et al. 2023).

Over the past two decades, there has been tremendous growth and expansion of emotional labor as a focal area of study in occupational health research (Gabriel et al. 2023; N. M. Humphrey 2023; Lee and Madera 2019). Wharton’s (2009) review offers a foundational synthesis of sociological approaches to emotional labor, and draws attention to the fragmentation between individual-level experiences and occupational analyses within the literature. Building on this early work, scholars from various disciplines have worked toward conceptualizing emotional labor as a dynamic and integrated process, where the occupational requirement for emotional labor informs the internal psychological process of emotion regulation—the mental and emotional effort expended to fulfill these requirements (Grandey and Gabriel 2015; Grandey and Melloy 2017). For this reason, some studies have come to consider both the job or occupational-level requirements of emotional labor and the emotion regulation process simultaneously in conceptual and analytical models. This sort of integrated model is particularly important for research that is focused on examining the health consequences of emotional labor. Such an analytical approach is beneficial for work and stress researchers because it allows for a more in-depth examination of how workers manage their emotions in front-line positions as emotional labor requirements vary in intensity. This has been a key consideration for recent studies focused on the health consequences of emotional labor performed by frontline service workers during the COVID-19 pandemic (Auger and Formentin 2021; O’Neil et al. 2023; Salvesen and Berg 2021).

### Emotional Labor Display Rules

The occupational requirement of emotional labor is guided by display rules, which are defined by Rafaeli and Sutton (1987) as the organizational norms and guidelines that dictate which emotions workers should express or suppress during service interactions. While Hochschild’s (1983) early work emphasized that emotional labor is a singular construct, most scholars agree that display rules can be categorized into positive and negative dimensions. Positive display rules involve the expression of warmth, enthusiasm, and friendliness, commonly seen in customer-facing roles (Diefendorff et al. 2006; Gabriel et al. 2023). Negative display rules focus on suppressing frustration, anger, or sadness, essential in high-stress environments such as healthcare and law enforcement (Glomb and Tews 2004; Grandey et al. 2005). Despite this distinction, display rules serve as antecedents that shape how workers engage in the emotional labor process (Ashforth and Humphrey 1993; Brotheridge and Lee 2002).

Display rules are deeply embedded in the occupational and organizational contexts where they operate, often reflecting broader cultural norms and expectations (Diefendorff et al. 2011; Grandey 2000). For instance, in caregiving professions like mental health services, positive display rules are intrinsic to fostering trust and therapeutic relationships, aligning with workers’ professional identities (Lee and Madera 2019; Wharton 2009). Conversely, roles in law enforcement or emergency response may emphasize negative display rules to maintain composure and authority in high-pressure situations (Erickson and Grove 2008; Grandey and Melloy 2017). These variations highlight the importance of understanding display rules not as static requirements but as dynamic expectations that interact with individual and situational factors. This contextual interplay shapes the beginning of the emotional labor process (Grandey and Melloy 2017).

### Emotion Regulation Strategies

Conceptually, the performance of emotional labor is facilitated by a process of emotion regulation, which occurs on an internal psychological level. In her original work, Hochschild (1983) identified two main strategies through which emotions are regulated in the context of emotional labor. The first is *surface acting*—where individual workers engage in a masked display of emotions that involves internally faking and suppressing their own felt emotions to achieve the display rule requirements of their work role. When this emotion regulation strategy is used, workers are not actually feeling the emotion they are displaying outwardly. For example, during the COVID-19 pandemic, many retail workers described feeling angered and frustrated when confronting maskless customers but nevertheless put on a smile to provide the service experience expected of them (Stix 2020). The alternative emotion regulation approach is *deep acting*, where individuals actively work to change their perception or feelings—in the moment—to align more genuinely with their emotional displays. In contrast to surface acting, deep acting is a more complex process comprised of cognitive-based strategies like perspective-taking, cognitive reappraisal, self-talk or mindfulness (Grandey 2000; Grandey and Melloy 2017). For example, a library staff member assisting a visibly upset patron may engage in deep acting through perspective-taking and will try to understand the patron’s needs or what they are looking for and will respond with empathy to provide appropriate support (Rodger and Erickson 2021).

While much is known about the ways in which surface acting and deep acting are performed, there are inconsistencies in research examining the health outcomes of emotion regulation (Groth et al. 2019; Judge, Woolf, and Hurst 2009). Surface acting is most consistently found in emotional labor research to be associated with negative health outcomes like increased emotional exhaustion, reduced job satisfaction and physiological stress responses (Brotheridge and Lee 2003; Gabriel et al. 2015; Grandey 2000). These consequences have been demonstrated across a wide range of occupations and occupational contexts (Hülsheger and Schewe 2011). Of particular importance is the link to emotional dissonance (Hochschild 1983)—a core concept in emotional labor research that represents the discrepancy between the emotions that a person displays in a given situation and the emotions that they actually feel. Emotional dissonance is considered a negative emotional and cognitive acute state for workers, because over time, it can lead to more chronic health conditions like burnout, psychological distress, and emotional exhaustion (Brotheridge and Grandey 2002; Brotheridge and Lee 2002; Zapf et al. 1999). Emotional dissonance is most often tied to surface acting, and this is because faking and suppressing one’s emotions leads to a discrepancy between felt and expressed emotions (Brotheridge and Lee 2002; Lewig and Dollard 2003).

In contrast, deep acting is considered by most scholars to be the emotion regulation strategy associated with better health outcomes. Grandey (2000) argues that deep acting, compared to surface acting, is a more effective and authentic form of emotion regulation. However, despite these arguments, deep acting does not consistently produce the findings to support this argument. In some studies, deep acting is associated with better health and well-being in comparison to surface acting, while in others, findings are null (Diefendorff and Richard 2003; Johnson and Spector 2007). Among the studies that find deep acting to be associated with positive health outcomes, it is argued that it serves as a protective psychological coping mechanism for workers. As a protective factor, deep acting is considered by researchers to be a psychological resource that buffers the negative effects of emotional labor (Brotheridge and Lee 2002; Zapf et al. 1999). Through deep acting, individuals are aligning their inner feelings with the required emotional display, which results in less emotional dissonance and greater perceived authenticity. From a conservation of resources (COR) perspective, deep acting has the potential to offset the mental and emotional resource loss from emotional labor by leading to resource gain (e.g., positive social feedback and genuine affective experiences) (Huang et al. 2015). While limited, some studies have found that deep acting is associated with worse mood (i.e. affectivity) (Judge et al. 2009).

If deep acting is considered a healthy emotion regulation strategy, why does it lead to inconsistent research results? One potential explanation is that there is more variation regarding the ways in which deep acting operates in emotional labor-intensive work. We argue that this is in part due to two overarching contextual and antecedent factors, (1) there is *much more* variability in the way that deep acting can be performed in comparison to surface acting (e.g., cognitive reappraisal and perspective taking) and (2) successful deep acting is contingent on the broader situational/occupational context (e.g., display rules). It is reasonable to believe that deep acting in response to suppressing negative display rules requires much more effort compared to deep acting in response to positive display rules. Hochschild (1983) argued that there are costs to both surface acting and deep acting and that over time the effort expended from regulating one’s emotions can lead to feelings of inauthenticity (alienation), emotional exhaustion and loss of personal identity.

Following Hochschild’s work, subsequent research refined conceptualizations of emotional labor by incorporating psychological models such as Gross’s (1998) early framework of emotionregulation to better understand emotional management in workplace contexts (Grandey 2000; Mikolajczak et al. 2009; Totterdell and Holman 2003). From this perspective, deep acting is recognized as a multidimensional strategy encompassing a range of antecedent-focused approaches—including cognitive reappraisal, attentional deployment, and perspective-taking— which aim to alter emotional responses before they fully develop (Grandey 2000; Gross 1998). Recent research suggests that the psychological costs and benefits of deep acting likely vary depending on the strategy employed (Alabak et al. 2020; Mann, Shane, and O’Brien 2024).

Although antecedent-focused emotion regulation strategies tend to be less psychologically damaging than surface acting (Brotheridge and Lee 2002; Gross 1998), they still require substantial mental and emotional effort, particularly when individuals must regulate strong negative emotions (Mikolajczak et al. 2009). In such cases, the cognitive demands of deep acting are likely heightened, and positive affective experiences that might otherwise compensate for regulatory effort are less accessible (Alabak et al. 2020). Grandey and Melloy’s (2017) revised model further emphasizes that deep acting may not be uniformly beneficial; rather, its outcomes depend on the emotional context in which regulation occurs.

Prior research has also shown that the consequences of emotional labor strategies may depend on the structure of the occupational environment. For example, Leidner’s (1993) comparative ethnographic study found that surface acting may be functional in highly routinized service roles, such as fast-food work, while deep acting may be more suitable in relational occupations like insurance sales, where authenticity and trust-building are central. This underscores the need to consider how occupational requirements interact with emotion regulation strategies in shaping psychological outcomes. We argue that the cognitive and emotional demands of deep acting, particularly under negative display rule conditions, may help explain why its relationship to mental health outcomes is inconsistent in emotional labor-intensive occupations. Earlier research on emotional labor in human service work has shown that managing agitated emotional states—such as anger, irritation, and nervousness—is more strongly associated with burnout and inauthenticity than managing other types of emotions, including sadness or cheerfulness (Erickson and Ritter 2001). These findings suggest that the psychological consequences of emotion regulation vary depending on the type of emotion being managed. This work further supports the notion that the psychological impact of emotion regulation is not uniform but may be contingent on the type of emotion being managed and the broader occupational context. Given the literature noted above, we draw from emotional labor theory and research to make the following hypotheses:

**Hypothesis 1:** Frontline workers who perceive higher emotional labor requirements in their job roles (positive and negative display requirements) will report higher levels of generalized anxiety, poor self-rated mental health and anger.
**Hypothesis 2:** Frontline workers who report engaging in surface acting more frequently will report higher levels of generalized anxiety, poor self-rated mental health and anger.
**Hypothesis 3:** Workers who report engaging in deep acting more frequently will report lower levels of generalized anxiety, poor self-rated mental health and anger.
**Hypothesis 4:** Emotion regulation will moderate the association between positive emotional labor display rule requirements and mental health, such that emotional labor will be more detrimental among workers who surface act and will be less detrimental among workers who deep act.
**Hypothesis 5:** Emotion regulation will moderate the association between negative emotional labor display rule requirements and mental health, such that negative display rule requirements will be detrimental among workers who engage in both surface acting and deep acting.

### Methodological Approaches to Emotional Labor and the Current Case Study

To-date, there has been a wide range of conceptual models and measurement of emotional labor across various studies and disciplines (Glomb and Tews 2004; Grandey et al. 2013; Simões, Gondim, and Puente-Palacios 2023). While some researchers have focused on self-reports of emotional labor requirements at the individual-level (Buckner and Mahoney 2012; Kruml and Geddes 2000), others have adopted a more objective approach, examining emotional labor requirements at the occupational-level (Bhave and Glomb 2016; Singh and Glavin 2017). Research using individual-level measures are typically focused on capturing self-reports of the frequency with which emotional labor is typically performed in an average workday as well as workers’ perceptions of the emotional labor requirements in their job role (Brotheridge and Lee 2003; Glomb and Tews 2004; Hong and Kim 2019). These studies tend to be smaller in scale and adopt a case study approach for examining workers’ subjective perceptions of display rules—a concept defined by Arlie Hochschild (1983) as the formal and informal rules that dictate how workers should express or suppress their emotions to meet organization and service-related expectations.

In contrast to self-report measures, a more recent development in emotional labor research has been the expansion of studies that use occupational-level measures of emotional labor requirements. In contrast to self-reports, display rules are captured through expert and job incumbent ratings of emotional requirements that are aggregated at the occupational-level. For example, Singh and Glavin (2017) used the Occupational Information Network Database (O*NET)—a database containing occupational information and characteristics from a comprehensive set of occupations in the United States—to construct a six-item composite scale representing the degree of emotional labor requirements associated with an occupation. A similar approach has been to measure display rules at the work-unit level (Diefendorff et al. 2011), where norms around emotional displays are constructed by different work groups within an organization (e.g., nursing units in a hospital). While occupational-level and unit-level studies are still limited in scope, researchers have argued that objective measures of emotional labor are less biased and provide the opportunity to examine emotional labor requirements on a much broader scale across many occupations and work contexts.

Despite the inconsistency in methodological approaches to measuring emotional labor requirements, there is value in both occupational-level and individual-level measurement strategies. Most researchers choose a measurement strategy that closely aligns with their research questions. While individual-level measures of emotional labor requirements may be subject to bias, or influenced by personality traits, it can be a useful measurement strategy for case study research that involve an in-depth examination of a select few occupations. For example, measures that capture emotional labor requirements as perceived negative and positive display rules, can be effective proxies for situational or antecedent factors that may influence how effective surface acting and deep acting are as emotion regulation strategies. Taken together, these measures allow for a more nuanced understanding of emotion regulation strategies in specific occupational contexts.

The current study adopts this approach for the purposes of capturing frontline workers’ individual-level experiences of performing emotional labor during the COVID-19 pandemic. We argue that despite its limitations, self-reported measures of emotional labor requirements can be useful for research examining unique and uncommon circumstances like the pandemic. We argue that alternative approaches would not have accurately captured the complex dynamics (e.g., frequency, intensity, and situational experiences) of emotional labor in frontline work during this period of time.

### The Frontline Occupational Context

During the COVID-19 pandemic, news articles were among the first to highlight the significant increase in emotional demands faced by human service workers, including occupations like library work that were not typically expected to face such challenges (Beattie 2021; Hune-Brown 2023). This was later echoed in studies documenting the emotional dissonance and burnout experienced by library staff, healthcare workers, and social service workers (Dowrick et al. 2021; Holmes et al. 2021; Rodger and Erickson 2021). Community mental health organizations experienced a sharp increase in demand for services related to homelessness and substance use during the pandemic (Canadian Mental Health Association [CMHA] 2022). In Canada, the CMHA reported that this surge in demand placed significant emotional and physical strain on mental health and addictions workers, contributing to heightened rates of burnout, compassion fatigue, and staff turnover (CMHA 2022). Many workers struggled to maintain their personal well-being while addressing the escalating and complex needs of their clients during this period (CMHA 2022).

Library workers, though not traditionally viewed as frontline responders, faced unique and amplified emotional labor challenges during the pandemic. Public libraries expanded their roles significantly to address heightened community needs, such as providing food banks, technology lending, and virtual programming to support isolated or underserved populations (Roberts and Clarke 2024). These roles often required providing social and emotional support to at-risk and vulnerable populations, further amplifying the emotional labor demands on library staff (Dalmer and Griffin 2022; Stevenson 2021). Emotional labor demands were heightened as workers enforced public health measures, frequently encountering incivility and, in some cases, violence from the public (Robinson, Ruthven, and McMenemy 2022). This exposure to distressing situations, such as overdoses or managing difficult patron interactions, contributed to chronic stress, burnout, and trauma among library staff, underscoring the critical need for trauma-informed support within these organizations (Grimes 2024; Wahler 2023).

These challenges may be most salient for mental health and addictions workers and library workers who support vulnerable populations in their communities, given that the pandemic exacerbated existing health disparities and vulnerabilities, resulting in an increased demand for services. While previous studies have provided critical insights into the emotional toll of frontline work during the pandemic, they often focus on either the emotional labor demands or their consequences in isolation. Our study builds on this literature by employing an integrated analytical model that simultaneously examines emotional labor requirements (positive and negative display rules) and emotion regulation strategies (deep and surface acting). By exploring the interaction between these components, this study provides a more nuanced understanding of how specific emotional labor processes affect mental health outcomes, thereby addressing gaps in existing research and contributing new insights into the emotional dynamics of frontline work during this unprecedented period.

In this study, we draw on a sample of frontline workers from local community organizations in mental health and addictions and library work in Ontario Canada during the early stages of the COVID-19 pandemic. As some studies have demonstrated, COVID-19 exacerbated emotional labor and frontline human service workers’ engagement in deep acting. (Pace, Sciotto, and Russo 2022). Therefore, this is an important consideration for research that seeks to address the inconsistencies on the health benefits of deep acting in emotional labor research. In addition, we use a self-reported measure of emotional labor requirements that enables us to examine positive and negative display rules separately. These measures are used to capture antecedent situational factors that may influence how effective deep acting is as an emotion regulation coping mechanism. Positive and negative display rules represent distinct dimensions of emotional labor requirements. By measuring them separately, researchers can differentiate between the effects of conforming to positive display rules (e.g., expressing positive emotions) and the effects of suppressing negative emotions in line with negative display rules. While previous research has aided our understanding of surface acting and deep acting, researchers have not explicitly focused on examining why theoretical expectations do not align with study results when deep acting is examined as a predictor for positive health outcomes.

## Methods

### Study Design and Data

To examine our hypotheses, we used data from the 2021 Emotions Matter Survey (EM), which constitutes a community-engaged investigation into emotional labor within the domains of mental health and addictions workers and library workers in Canada. The EM survey collected individual-level data online (via Qualtrics) from a demographically diverse group of working adults, specifically drawing samples from 11 Canadian Mental Health Association branches (CMHA) and four Public Library systems located across Ontario, Canada. Sample selection involved separate simple random sampling from both CMHA branches and library systems. The study’s participants were restricted to full-time employees of these community organizations who were actively engaged in public-facing work. The survey achieved a response rate of 56 percent from CMHA and 62 percent from the public libraries, culminating in a total sample size of 854 responses.

### Dependent Measures

#### Anxiety

Anxiety is measured using the Composite International Diagnostic Interview Screening Scales (CIDI-SC) for anxiety. The CIDI-SC are short screening scales developed based on the World Health Organization’s (WHO) Composite International Diagnostic Interview (CIDI). The measure has been shown to have good psychometric properties and concordance with the full CIDI. Items were summed to create a scale (alpha = .92).

#### Anger

We measure anger with five items taken from the 2005 Work Stress and Health Study (WSH) (Schieman and Reid 2009). Respondents were asked on how many days in the past seven days they “felt annoyed or frustrated,”“felt angry,”“felt critical of others,”“yelled at someone about something,” and “lost your temper.” Items were averaged such that higher numbers equal a greater amount of anger reported. Results can be interpreted as the number of days in the previous week. Items showed good internal consistency (alpha = .85).

#### Self-reported mental health

Self-reported mental health was measured using a single item also taken from the 2005 WSH. Respondents were asked how they would rate their mental health at the present time with response options: (1) Poor; (2) Fair; (3) Good; (4) Very good; and (5) Excellent. Responses were coded as 1 for poor and fair, and as 0 for good, very good, and excellent.

### Focal Independent Measures

#### Emotional labor requirements

Emotional labor was measured using the Emotion Work Requirements Scale (Best, Downey, and Jones 1997; Brotheridge and Grandey 2002). The measure contains seven items with a 5-point response scale (1 = not at all required, 5 = always required). *See Appendix B for full list of items*. Items asked respondents how often they are required to manage emotions at work. Based on factor analysis from preliminary studies, the items load on two factors: (1) positive display rules (e.g., expressing feelings of sympathy) that focus on requirements to display positive emotions at work; and (2) negative display rules (e.g., hiding anger or disapproval) that focus on requirements to hide negative emotions at work (Jones and Best 1995). Dividing the items in this way shows good internal consistency with a scale reliability coefficient of .81 for positive display rules and .84 for negative display rules. *See Appendix C for factor analysis results*.

#### Emotion regulation

Emotion regulation was addressed with two variables: deep acting and surface acting. These items were a part of the Emotional Labor Scale (Brotheridge and Lee 1998, 2002). Surface acting refers to faking emotions to meet work requirements while deep acting refers to a process of modifying feelings to meet work display rules. Surface acting items asked respondents the extent to which they (1) resist expressing their true feelings; (2) pretend to have emotions that they don’t really have; and (3) hide their true feelings about a situation. Items were combined by calculating row means with a resulting scale reliability coefficient of .86. Deep acting items asked respondents the extent to which they (1) make an effort to actually feel the emotions they need to display to others; (2) try to actually experience the emotions they must show; and (3) really try to feel the emotions they have to show as part of their job. Response options consisted of “none of the time,”“a little of the time,”“some of the time,”“most of the time,” and “all or almost all of the time.” Items were combined by summing across items with a resulting scale reliability coefficient of .94.

### Important Covariates

All models included the following control variables.

To assess *job autonomy*, respondents were asked how often someone else decides how they do their work, with response options “never,”“rarely,”“sometimes,” and “frequently.”

*Job authority* was measured by asking respondents whether they have the authority to hire and fire others with response options “Yes” and “No.”

*Client contact* was assessed by asking respondents if they have contact with clients as a part of their job with response options “Yes” and “No.”

*Demographic and social controls included* sex coded 0 “Male,” 1 “Female,” and 2 “Other”; Work site coded 0 “library” and 1 “mental health workers,” age in years, and race coded 0 “White” and 1 “non-White.”

### Plan of Analyses

To address missing data, multiple imputation via chained equations (MICE) was applied (Rubin 1987). Imputation models included all variables across all analyses and produced 10 imputed datasets. Analysis was conducted in two steps. First, main effects models were run on each of the three outcomes using OLS and logistic regression as appropriate. Next, interaction models testing interactions between emotional labor requirements (i.e., positive and negative display rules) and emotion regulation strategies (i.e., surface and deep acting) were run to determine whether the effect of emotional labor requirements on the mental health outcomes were contingent on the type of emotion regulation strategy applied. Only statistically significant interactions were retained in final analyses and presentation of results.

### Additional Analyses for Interactions Using Average Marginal Effects for Logistic Regression

For our binary outcome of self-reported mental health for which we use logistic regression, we have provided additional analyses. This is due to questions regarding the accuracy of interaction coefficients in analyses with a binary outcome since the interaction coefficients estimated in logistic regression analyses do not necessarily indicate significant conditional effects (see Allison 1999; Long and Mustillo 2021; Mize 2019). We calculated the average marginal effects for the slope of the line for negative display rules on the probability of poor or fair self-reported mental health at various levels of deep acting, as recommended by Mize (2019). We then used Wald tests to determine statistically significant increases in the predicted probability of reporting poor or fair mental health for each unit increase in negative display rules at each of the selected levels of deep acting. We presented these findings in both Figure 3 and Appendix A (Table A3).

## Results

Table 1 presents sample demographics and missing data information. All variables included in our analyses had less than five percent missing with the exception of age which had 12.65 percent missing. Little’s test of missing completely at random indicate this condition is met in order to proceed with the MICE (Li 2013). The sample is 80 percent female with an average age of approximately 41 years. 72 percent of the sample self-identified as White with 64 percent being mental health professionals and the remaining 36 percent being public library professionals.

**Table 1.** Descriptive Statistics of Sample.

Variables	Mean values or proportions (Std. errors)	Range	Proportion missing
*Outcomes*			
Anxiety	7.98 (0.15)	0–16	1.87%
Anger	2.04 (0.05)	0–35	1.99%
Self-Rated Mental Health—Fair/Poor	0.43 (0.02)		1.76%
*Focal Independent Variables*			
Surface Acting	5.38 (0.10)	0–12	1.99%
Deep Acting	5.23 (0.12)	0–12	3.40%
Positive Display Rules	11.95 (0.11)	0–16	1.64%
Negative Display Rules	6.10 (0.11)	0–12	1.64%
*Demographic Variables*			
Female (vs Male/Other)	0.80 (0.01)		1.99%
Age (years)	41.19 (0.45)	18–68	12.65%
White (vs Other)	0.72 (0.02)		1.05%
*Work Context*			
Autonomy	1.33 (0.03)	0–3	2.93%
Authority (vs no authority)	0.09 (0.01)		3.75%
Client Contact (vs no contact)	0.74 (0.02)		4.80%
Library (vs Mental Health Professional)	0.64 (0.02)		0.00%

### Anxiety

Table 2 displays results for the OLS regression of anxiety on focal independent variables and controls. Model 1 displays the results from the main effect model. Results show partial support for Hypothesis 1. Initially, we find that a unit increase in negative display rules is associated with a significant increase in anxiety (*b* = 0.124, *p* < .05) and find no significant results for positive display rules. We also find that a unit increase in surface acting is associated with a significant increase in anxiety (*b* = 0.450, *p* < .001), supporting Hypotheses 2. However, there are no significant results for deep acting (Hypothesis 3).

**Table 2.** OLS Regression of Anxiety on Focal Independent Variables and Controls.

	Model 1	Model 2
*Emotional Labor*		
Positive Display Rules	.015 (.054)	−.006 (.054)
Negative Display Rules	.124* (.053)	−.044 (.075)
Surface Acting	.450*** (.052)	.430*** (.052)
Deep Acting	−.027 (.042)	−.236** (.078)
*Respondent Demographics*		
Gender		
Female	1.141** (.35)	1.163** (.345)
Other	1.545 (1.01)	1.522 (1.005)
Age	−.070*** (.012)	−.078*** (.012)
White^a^	.566 (.306)	.543 (.304)
*Work Context*		
Autonomy	−.373* (.163)	−.402* (.162)
Site		
Libraries^b^	.034 (.298)	.030 (.296)
Authority	.548 (.463)	.553 (.460)
Client contact	−.078 (.313)	−.052 (.311)
*Interactions*		
Negative Display Rules × Deep Acting	—	.036** (.011)
Constant	7.126*** (1.014)	8.519*** (1.109)
*R* ^2^	.233	.243

*Note*. *R*^2^ represents the mean across 10 imputations. Standard errors included in parentheses.

a vs other.

b vs mental health professionals.

* *p* < .05. **p < .01. ***p < .001.

In model 2, interactions between emotional labor requirements and emotion regulation strategies were tested (Hypotheses 4 and 5). Results show a significant interaction between negative display rules and deep acting (*b* = 0.036, *p* < .01). Figure 1 displays this result. An analysis of simple slopes shows that when deep acting is at its mean or one standard deviation above the mean, a unit increase in negative display rules resulted in a significant increase in anxiety (Table A1). No other significant interactions were observed among emotional labor variables and emotion regulation variables (see Appendix D for full models including non-significant interactions).

**Figure 1.**
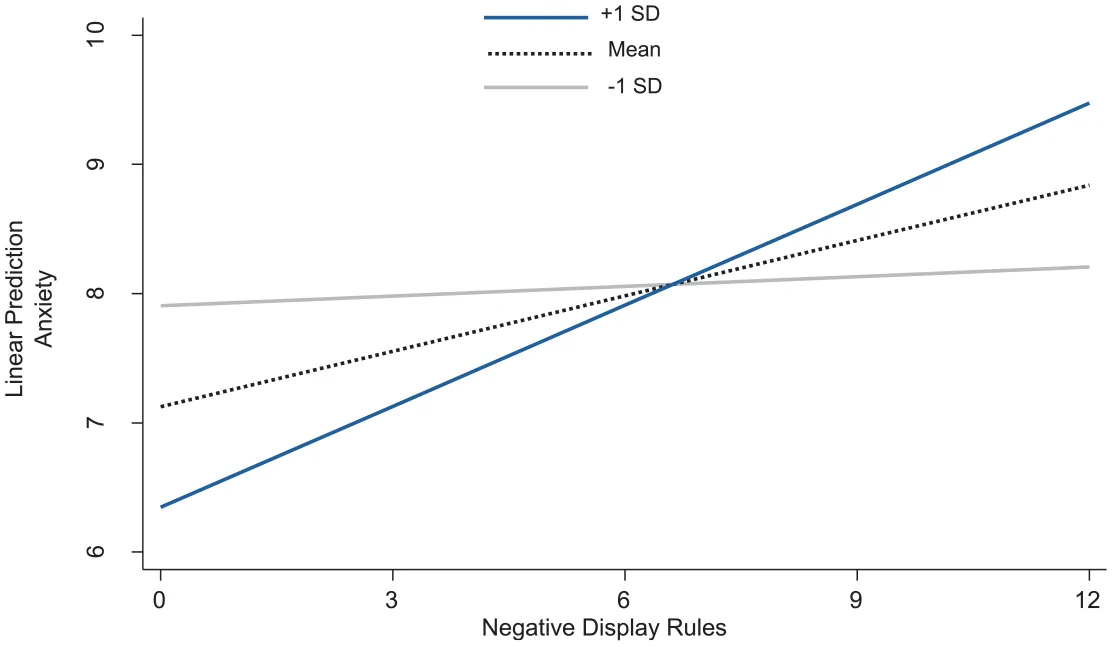
The association between negative display rules and anxiety at various points of deep acting.

### Anger

Table 3 displays results for the OLS regression of anger on focal independent variables and controls. Model 1 displays the results from the main effect model. We find no support for Hypotheses 1 and 3. However, similar to anxiety, results suggest that a unit increase in surface acting is associated with a significant increase in anger (*b* = 0.164, *p* < .001), providing support for Hypothesis 2. Unlike anxiety, negative display rules did not initially appear to have an effect. However, we need to take deep acting into consideration, as we do in model 2.

**Table 3.** OLS Regression of Anger on Focal Independent Variables and Controls.

	Model 1	Model 2
*Emotional Labor*		
Positive Display Rules	−.036 (.019)	−.042* (.019)
Negative Display Rules	.033 (.019)	−.024 (.026)
Surface Acting	.164*** (.018)	.158*** (.018)
Deep Acting	.017 (.015)	−.053* (.028)
*Respondent Demographics*		
Gender		
Female	.159 (.123)	.167 (.123)
Other	−.283 (.360)	−.291 (.360)
Age	−.009* (.004)	−.010* (.004)
White^a^	.256* (.109)	.249* (.108)
*Work Context*		
Autonomy	−.108 (.060)	−.118* (.060)
Site		
Libraries^b^	−.211* (.108)	−.212* (.108)
Authority	.278 (.166)	.280 (.165)
Client contact	.090 (.120)	.096 (.120)
*Interactions*		
Negative Display Rules × Deep Acting	–	.012** (.004)
Constant	1.508*** (.358)	1.979*** (.389)
*R* ^2^	.162	.171

*Note*. *R*^2^ represents the mean across 10 imputations. Standard errors included in parentheses.

a vs other.

b vs mental health professionals.

* *p* < .05. **p < .01. ***p < .001.

In model 2, interactions between emotional labor requirements and emotion regulation strategies were tested. Similar to anxiety, a significant interaction between negative display rules and deep acting was observed (*b* = .012, *p* < .01). Figure 2 illustrates this result. An analysis of simple slopes revealed that when deep acting is at its mean or one standard deviation above the mean, a unit increase in negative display rules was associated with a significant increase in anger (Table A2). No other significant interactions were observed and so were not included in the models (see Appendix D for full models including non-significant interactions).

**Figure 2.**
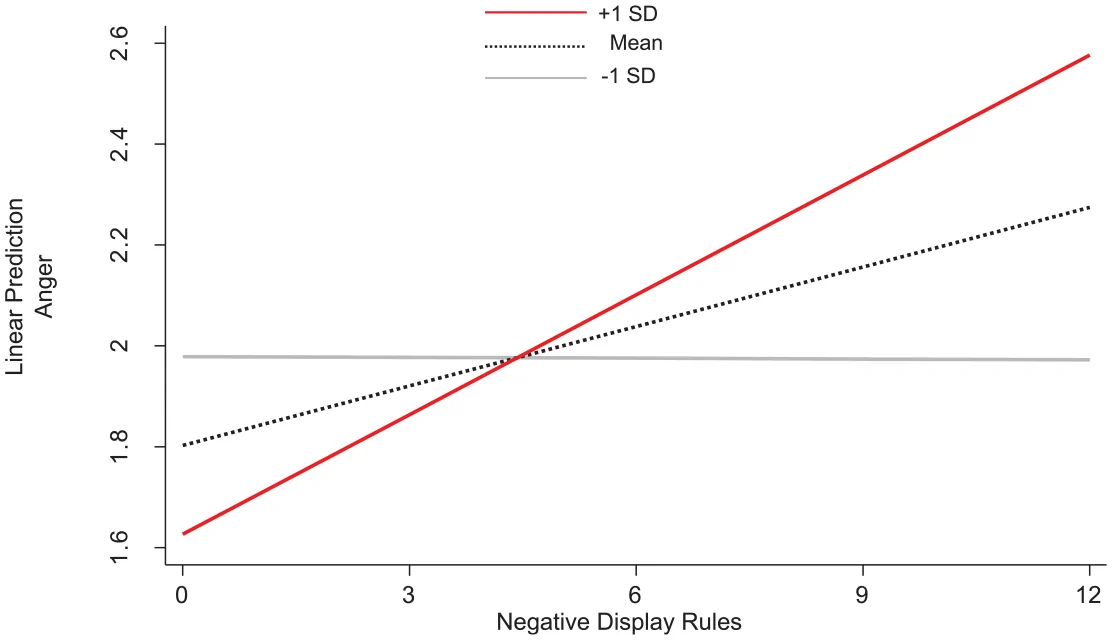
The association between negative display rules and anger at various points of deep acting.

### Self-Reported Mental Health

Table 4 shows results of the logistic regression of self-reported mental health on focal independent variables and controls. The main effect model (Model 1) initially shows that a unit increase in negative display rules is associated with an increase in the log odds of self-reporting mental health as fair or poor (*b* = .081, *p* < .01). This is also the case for surface acting with a unit increase in surface acting associated with an increase in the log odds of self-reporting mental health as fair or poor (*b* = .222, *p* < .001). We also find that a one-unit increase in deep acting is associated with an increase in the log odds of reporting fair or poor self-rated mental health (*b* = .048, *p* < .05), contrary to Hypothesis 3, which predicted a protective association.

**Table 4.** Logistic Regression of Self-Rated Mental Health on Focal Independent Variables and Controls.

	Model 1	Model 2
*Emotional Labor*		
Positive Display Rules	−.052 (.033)	−.059 (.033)
Negative Display Rules	.081** (.081)	.015 (.046)
Surface Acting	.222*** (.032)	.218*** (.032)
Deep Acting	.048* (.025)	−.035 (.052)
*Respondent Demographics*		
Gender		
Female	.221 (.206)	.227 (.207)
Other	1.426* (.730)	1.398* (.731)
Age	−.026*** (.007)	−.027*** (.007)
White^a^	.184 (.182)	.179 (.183)
*Work Context*		
Autonomy	−.177 (.095)	−.191* (.096)
Site		
Libraries^b^	−.695*** (.179)	−.704*** (.180)
Authority	.091 (.270)	.090 (.270)
Client contact	.166 (.190)	.170 (.191)
*Interactions*		
Negative Display Rules × Deep Acting	–	.013* (.007)
Constant	−.536 (.600)	.016 (.668)
Pseudo *R*^2^	.138	.141

*Note*. Pseudo *R*^2^ represents the mean across 10 imputations. Standard errors included in parentheses.

a vs other.

b vs mental health professionals.

* *p* < .05. **p < .01. ***p < .001.

Model 2 shows the result for the interaction between negative display rules and deep acting. Unlike anxiety and anger, the interaction is only marginally significant for self-reported mental health, but the pattern is consistent with simple slopes revealing that when deep acting is at its mean or one standard deviation above the mean, a unit increase in negative display rules was associated with an increased log odds of self-reporting mental health as fair or poor (Table A3). This result is displayed in Figure 3. No other significant interactions were observed and were therefore not included in the model (see Appendix D for full models including non-significant interactions).

**Figure 3.**
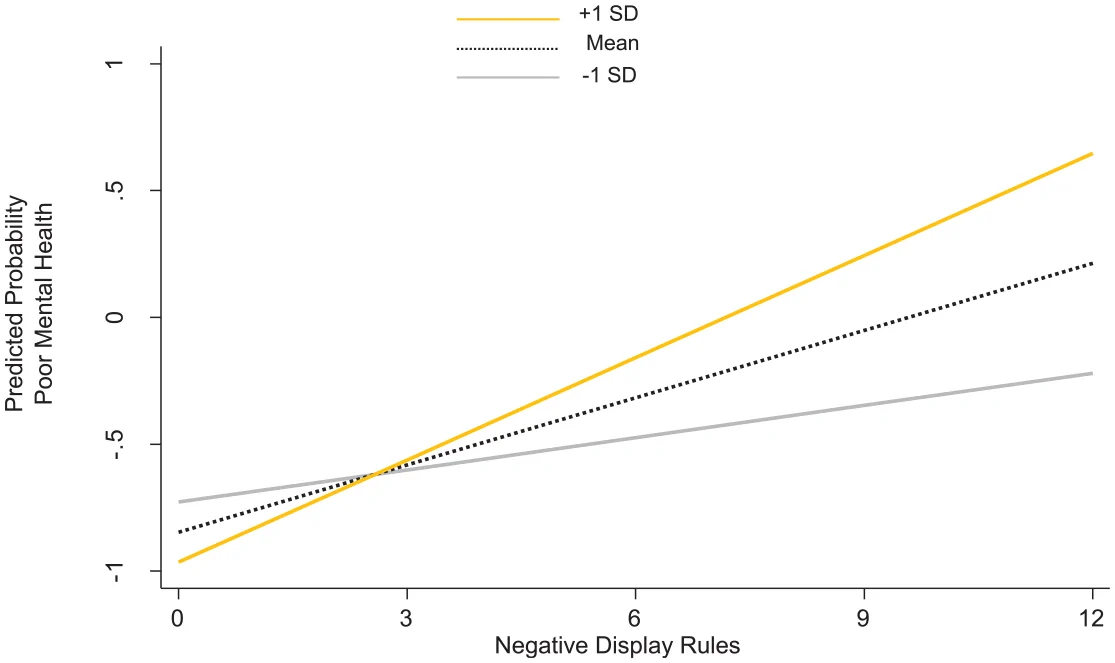
The association between negative display rules and the predicted probability of reporting poor/fair mental health at various points of deep acting.

## Discussion

In the evolving landscape of emotional labor research, adopting an integrated approach to examine the consequences of emotional labor is not only innovative but has also been considered necessary by researchers in the area. This article uniquely contributes to the literature by simultaneously considering the interplay of self-reported emotional labor job requirements and emotion regulation strategies, a perspective that has been underexplored in previous research. Furthermore, by examining both internalizing (e.g., anxiety) and externalizing (e.g., anger) outcomes, this study adopts a comprehensive approach of capturing diverse dimensions of mental health to fully understand the impact of emotional labor (Aneshensel 2005). While deep acting is often considered a healthier emotion regulation strategy compared to surface acting, the literature reveals inconsistent results regarding its impact on mental health. Some studies indicate positive health outcomes associated with deep acting, while others find null or even negative effects (Aziz, Widis, and Wuensch 2018; Huppertz et al. 2020). This inconsistency points to a complex underlying mechanism, possibly influenced by the variability in how deep acting is performed and the specific situational and occupational contexts in which it is employed (Alabak et al. 2020). Our study sheds light on this inconsistency using self-reported measures of emotional labor and emotion regulation in the unique context of front-line work. This methodological choice captures the nuanced, subjective experiences of workers, enabling a more authentic and comprehensive understanding of the psychological impact of emotional labor and the varying impacts of deep acting. By integrating these elements, this article aims to clarify the complex mechanisms by which emotional labor influences mental health outcomes, marking a significant advancement in the field.

Our analytical approach is particularly critical in addressing the multifaceted nature of emotional labor, acknowledging that its impact on mental health is not merely additive but often conditional and context specific. Adopting this analytical technique aligns our study with recent academic discourse that advocates for a more integrated and dynamic examination of emotional labor (e.g., Grandey and Gabriel 2015; Grandey and Melloy 2017). The objective of our analysis was to systematically examine the relationship between emotional labor, emotion regulation strategies, and their subsequent impact on mental health among frontline workers. Using ordinary least squares (OLS) and logistic regression analyses, we examined both the individual and interactive effects of various facets of emotional labor—specifically, negative and positive display rules—and emotion regulation strategies, namely, surface and deep acting, on key mental health outcomes including anxiety, anger, and self-reported mental health.

### Display Rules and Mental Health

The following sections unpack and elaborate on two key findings: the effects of surface acting and deep acting on mental health outcomes; and the contingent effect of deep acting on mental health outcomes on negative display rules.

Our results revealed a clear relationship between the requirements of emotional labor, particularly its negative dimension, and heightened anxiety such that greater reported negative display rules resulted in heightened anxiety on average, validating our first hypothesis. In contrast, positive display rules did not significantly affect anxiety levels, offering only partial confirmation of our hypotheses. Interestingly, negative display rules had no marked influence on anger. While the literature suggests that negative emotions narrow individual’s thought-action processes to specific actions (e.g. attack and flee), it may not uniformly amplify all negative emotions such as anger (Frederickson and Branigan 2005). This could indicate that the regulation of anger may be context-dependent or influenced by factors beyond the scope of our current study. Further exploration into the broader consequences for mental health indicated that negative display rules also exacerbated the odds of individuals reporting their mental health as fair or poor. This finding underscores the pervasive effect of negative emotional labor requirements on overall well-being.

The distinction between the effects of negative and positive display rules warrants closer examination. Negative display rules require workers to suppress emotions such as frustration, anger, or sadness, which often leads to emotional dissonance—a misalignment between felt and expressed emotions (Morris and Feldman 1996; Wharton 1999). This dissonance is known to have deleterious effects on mental health, including heightened anxiety and burnout (Brotheridge and Grandey 2002; Grandey, Rupp, and Brice 2015). By contrast, positive display rules, which mandate the expression of empathy, warmth, and enthusiasm, may align more closely with intrinsic occupational norms (Diefendorff, Richard, and Croyle 2006; R. H. Humphrey, Ashforth, and Diefendorff 2015). This is known to be particularly evident in caregiving occupations, like mental health work, where these emotional expressions are central to professional identity. When positive display rules align with intrinsic occupational expectations, they may reduce the psychological strain associated with these rules, which might explain their lack of significant direct effects on mental health in our study.

Moreover, the occupational context plays a crucial role in shaping these outcomes. Mental health and addictions workers, for example, may find positive display rules to be a natural extension of their therapeutic interactions, where expressions of care and empathy are intrinsic to their professional identity (Lee and Madera 2019). Conversely, library workers, whose roles are less traditionally associated with caregiving, may encounter positive display rules as situational demands rather than intrinsic expectations. This difference in role alignment may contribute to the variation in how these rules impact mental health. Future research could explore how occupational norms and role expectations moderate the effects of positive display rules on mental health.

### Effects of Deep Acting and Surface Acting on Mental Health Outcomes

As expected, our findings confirm the anticipated negative psychological outcomes associated with surface acting such that greater reported surface acting resulted in worse mental health outcomes. This underscores the detrimental effects of the dissonance created when one’s internal feelings do not align with external expressions (Brotheridge and Grandey 2002; Grandey, Rupp, and Brice 2015). This pattern, consistent across outcomes, highlights the broader negative implications of certain emotional labor practices. Overall, these results lend strong support to the hypothesis that surface acting, characterized by altering one’s outward emotional displays without changing the underlying emotions, is harmful to emotional well-being.

Moreover, while we hypothesized that surface acting would moderate the association between emotional labor requirements and mental health outcomes (Hypotheses 4 and 5), no significant moderation effects were found. This suggests that surface acting may exert a consistently detrimental effect on mental health across different emotional labor contexts, rather than amplifying or weakening the effect of specific display rule requirements. In other words, regardless of whether workers are required to express positive or suppress negative emotions, the act of surface acting appears to impose a general psychological burden. These findings align with prior research suggesting that surface acting, by perpetuating emotional dissonance, functions as a broad occupational stressor with negative mental health implications (Bono and Vey 2005; Hülsheger and Schewe 2011). Future research may benefit from examining other potential moderators, such as job resources or organizational support, that could buffer the negative effects of surface acting on well-being.

Contrary to expectations, we found that greater engagement in deep acting was associated with a higher likelihood of reporting poor or fair self-rated mental health in the main effects model. This finding contradicts Hypothesis 3, which anticipated a protective association. No significant direct effects of deep acting were observed for our other psychological outcomes of interest. Taken together, these results are consistent with prior research highlighting the mixed effects of deep acting on mental health (Alabak et al. 2020; Huppertz et al. 2020). Our analysis of interaction effects further suggests that the psychological consequences of deep acting may be contingent on other occupational factors, such as the nature of emotional display rule requirements.

### Negative Display Rules and Deep Acting

Of particular note in our findings was the dependence of the effect of deep acting on mental health outcomes through negative display rules. More specifically, our findings showed that the effect of negative display rules tended to be worse at higher reported levels of deep acting, suggesting a complex interplay between these dimensions of emotional labor. Contrary to the commonly held view that deep acting is a healthier emotion regulation strategy (Grandey and Gabriel 2015), our findings suggest that when deep acting is high, it exacerbates the negative impact of adhering to negative display rules on mental health, leading to increased anxiety, anger, and a higher odds of self-reporting poor or fair health. This is not observed with positive display rules, indicating a distinct interplay between the type of emotional labor and the regulation strategy employed.

One plausible explanation for this pattern lies in the nature of deep acting itself. Deep acting involves an intensive internal process of aligning one’s genuine feelings with external expressions (Grandey and Sayre 2019; Wharton 2009). For instance, in frontline mental health and addictions work, professionals often encounter highly charged emotional situations where they must display calm and empathy despite potentially feeling overwhelmed or frustrated internally. When this deep acting process is applied in the context of adhering to negative display rules, such as maintaining a composed demeanor in the face of aggressive or distressing client behavior, it may require a substantial psychological effort. This effort to transform genuine negative emotions into the required positive outward expressions can be psychologically taxing. The internal conflict and effort of managing such emotional dissonance, while necessary for professional conduct, can lead to heightened emotional strain and, consequently, poorer mental health outcomes (Allard, Oliphant, and Lieu 2023; Clarke et al. 2021; Edward, Hercelinskyj, and Giandinoto 2017; O’Neil et al. 2023). In contrast, aligning one’s emotions with positive display rules through deep acting, such as genuinely cultivating empathy and understanding in therapeutic interactions, may be less taxing or even beneficial, as it does not involve the same degree of emotional conflict and dissonance (Lee and Madera 2019). This difference highlights the context-specific nature of emotional labor and its varying impacts on mental health, especially in high-intensity roles like those in mental health and addictions and library work.

Overall, these findings indicate that the efficacy and impact of deep acting as an emotion regulation strategy are highly contingent on the nature of the emotional labor demands, particularly the type of display rules. They underscore the importance of context-specific considerations in understanding the mental health implications of emotional labor and highlight the need for nuanced approaches in managing emotional labor, especially in high-stress occupational settings. This contributes to a more complex and holistic understanding of the interplay between emotional labor and mental health, advancing the discourse in occupational health research.

### Limitations

While our study contributes to the ongoing evidence on the impact of emotional labor on mental health outcomes, it is not without its limitations. First, the reliance on self-reported measures of emotional labor and emotion regulation strategies may be more susceptible to response biases (Grandey and Gabriel 2015). However, these have been shown to be more valuable for capturing individual’s perceptions which are vital for the study of emotions (Judge et al. 2009). Second, the study data was collected during the unique context of the COVID-19 pandemic. Therefore, the cross-sectional nature of this study could limit its generalizability to other settings or times. However, a longitudinal follow up is currently being conducted with the same participants to assess whether these patterns change over time. Future research could benefit from incorporating alternative measures of emotional labor that reflect occupational requirements rather than individual perceptions or employing longitudinal designs to assess the long-term impacts of emotional labor on mental health. There is also a need for further exploration into the variability in deep acting strategies and how they interact with different types of emotional labor requirements across diverse occupational settings. In addition, future research could explore other externalizing outcomes, such as hostility or aggression, and their interplay with internalizing dimensions like anxiety. This would build on the current study’s findings by investigating how these outcomes are influenced by the variability in emotion regulation strategies across occupational settings, thereby enhancing our understanding of the conditions under which emotional labor functions as a stressor.

## Conclusion

We argue that this study makes a significant contribution to research on the consequences of emotional labor, shedding light on the intricate relationship between emotional labor, emotion regulation, and mental health in the unique context of front-line work during the COVID-19 pandemic. The findings of this study underscore the complexity of emotional labor’s impact on mental health, particularly highlighting the nuanced role of deep acting when negative display rules are required. This research contributes to the ongoing academic discourse by offering a more integrated and dynamic perspective on emotional labor, challenging previous assumptions and encouraging a revaluation of the mechanisms through which emotional labor influences mental health.

As the landscape of work continues to evolve in a post COVID-19 context, especially in high-stress environments like front-line human service work, the insights from this study provide a foundation for future explorations and interventions aimed at enhancing the well-being of those on the front lines of service delivery. Understanding these interaction effects is essential for crafting targeted support mechanisms that acknowledge the multifaceted nature of emotional labor. This knowledge can guide community-engaged research and organizations in developing training programs, support systems, and organizational policies that not only address the challenges of emotional labor but also leverage its potential benefits for improving mental health and overall job satisfaction among employees.

## Appendix A

### Average Marginal Effects

**Table A1.** Average Marginal Effects from the OLS Regression of Negative Display Rules on Anxiety at Various Points of Deep Acting.

Level of deep acting	Average marginal effect
0	−0.044 (0.075)
2	0.027 (0.061)
4	0.099 (0.053)
6	0.170* (0.054)
8	0.241*** (0.063)
10	0.313*** (0.078)
12	0.384*** (0.096)

* *p* < .05. ***p* < .01. ****p* < .001.

**Table A2.** Average Marginal Effects from the OLS Regression of Negative Display Rules on Anger at Various Points of Deep Acting.

Level of deep acting	Average marginal effect
0	−0.024 (0.026)
2	0.001 (0.021)
4	0.024 (0.019)
6	0.048* (0.019)
8	0.073** (0.023)
10	0.097** (0.028)
12	0.121** (0.035)

* *p* < .05. ***p* < .01. ****p* < .001.

**Table A3.** Average Marginal Effects from the Logistic Regression of Negative Display Rules on Self-Reported Mental Health at Various Points of Deep Acting.

Level of deep acting	Average marginal effect
0	0.015 (0.046)
2	0.043 (0.037)
4	0.071* (0.031)
6	0.099* (0.032)
8	0.127* (0.039)
10	0.155* (0.050)
12	0.182* (0.062)

* *p* < .05. ***p* < .01. ****p* < .001.

## Appendix B

### Emotional Labor Scales

**Table B1.** Emotional Labor Requirement Items.

Scale	Item	Item number
Positive Display Rules	Reassure people who are distressed or upset	1
Positive Display Rules	Remain calm even when you are astonished	2
Positive Display Rules	Express feelings of sympathy (e.g., saying you “understand,” you are sorry to hear about something)	3
Positive Display Rules	Express friendly emotions (e.g., smiling, giving compliments, and making small talk)	4
Negative Display Rules	Hide your anger or frustration about something someone has done (e.g., an act that is distasteful to you)	5
Negative Display Rules	Hide your disapproval over something someone has done	6
Negative Display Rules	Hide your fear of someone who appears threatening	7

*Note*. Preamble for these items reads “On an average day at work, how frequently do you…?.”

## Appendix C

### Factor Analysis of Emotional Labor Items

**Table C1.** Eigenvalues of Emotional Labor Requirements from Factor Analysis.

Factor	Eigenvalue
Factor 1	2.27524
Factor 2	1.93323
Factor 3	0.5535

*Note*. Maximum likelihood factor method used.

**Table C2.** Rotated Factor Loadings for Emotional Labor Requirements (Varimax Rotation)

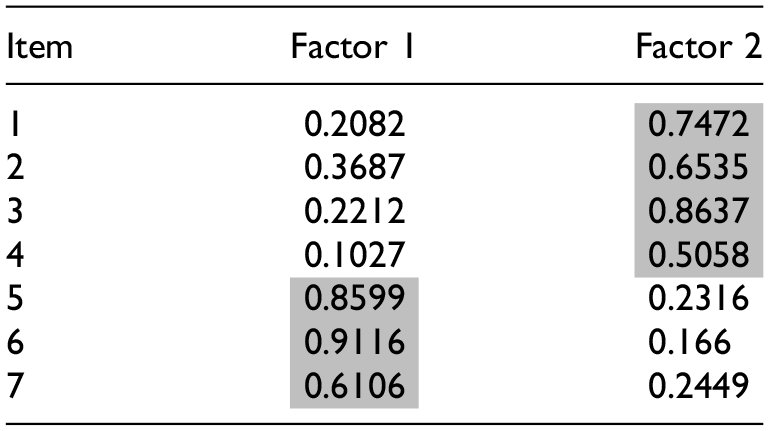

*Note*. Orthogonal varimax rotation used. Cells highlighted in gray indicate the factor loading.

Item numbers correspond to item numbers in Appendix B.

## Appendix D

### Supplementary Tables of Non-Significant Interaction Effects

**Table D1.** OLS Regression of Anxiety on Focal Independent Variables and Controls for Non-Significant Interactions.

	Model 1	Model 2	Model 3
*Emotional Labor*			
Positive Display Rules	.015 (.054)	.064 (.083)	−.110 (.085)
Negative Display Rules	.121 (.081)	.124* (.053)	.127* (.052)
Surface Acting	.445*** (.102)	.574** (.165)	0.441*** (.052)
Deep Acting	−.027 (.042)	−.025 (.042)	−.273* (.137)
*Respondent Demographics*			
Gender			
Female	1.142** (.348)	1.149** (.347)	1.130** (.346)
Other	1.546 (1.011)	1.571 (1.011)	1.525 (1.009)
Age	−.078*** (.012)	−.078*** (.012)	−.079*** (.012)
White^a^	.565 (.306)	.575 (.306)	.561 (.306)
*Work Context*			
Autonomy	−.372* (.163)	−.383* (.163)	−.377* (.163)
Site			
Libraries^b^	.034 (.298)	.030 (.298)	.039 (.297)
Authority	.549 (.463)	.537 (.463)	.552 (.462)
Client contact	−.070 (.313)	−.072 (.313)	−.033 (.313)
*Interactions*			
Negative Display Rules × Surface Acting	.001 (.013)	–	–
Positive Display Rules × Surface Acting	–	−.010 (.013)	–
Positive Display Rules × Deep Acting	–	–	.021 (.011)
Constant	7.149*** (1.077)	6.528*** (1.261)	8.688*** (1.302)
*R* ^2^	.233	.234	.237

*Note*. *R*^2^ represents the mean across 10 imputations. Standard errors included in parentheses.

a vs other.

b vs mental health professionals.

* *p* < .05. ***p* < .01. ****p* < .001.

**Table D2.** OLS Regression of Anger on Focal Independent Variables and Controls for Non-Significant Interactions.

	Model 1	Model 2	Model 3
*Emotional Labor*			
Positive Display Rules	−.036 (.190)	−.016 (.029)	−.035 (.031)
Negative Display Rules	.036 (.029)	.033 (.019)	.033 (.019)
Surface Acting	.168*** (.037)	.213*** (.060)	0.164*** (.019)
Deep Acting	.017 (.015)	.018 (.015)	.019 (.049)
*Respondent Demographics*			
Gender			
Female	.159 (.123)	.162 (.123)	.160 (.123)
Other	−.284 (.360)	−.273 (.360)	−.283 (.361)
Age	−.010* (.004)	−.010* (.004)	−.010* (.004)
White^a^	.257* (.109)	.260* (.109)	.257* (.109)
*Work Context*			
Autonomy	−.109 (.060)	−.112 (.163)	−.108 (.060)
Site			
Libraries^b^	−.211 (.108)	−.212 (.108)	−.211 (.108)
Authority	.277 (.166)	.274 (.166)	.278 (.166)
Client contact	.090 (.120)	.089 (.120)	.089 (.120)
*Interactions*			
Negative Display Rules × Surface Acting	−.001 (.004)	–	–
Positive Display Rules × Surface Acting	–	−.003 (.004)	–
Positive Display Rules × Deep Acting	–	–	−.001 (.004)
Constant	1.492*** (.381)	1.273** (.450)	1.496** (.466)
*R* ^2^	.162	.163	.162

*Note*. *R*^2^ represents the mean across 10 imputations. Standard errors included in parentheses.

a vs other.

b vs mental health professionals.

* *p* < .05. ***p* < .01. ****p* < .001.

**Table D3.** Logistic Regression of Self-Reported Mental Health (1 = Poor/Fair, 0 = Good, Very Good, Excellent) on Focal Independent Variables and Controls for Non-Significant Interactions.

	Model 1	Model 2	Model 3
*Emotional Labor*			
Positive Display Rules	−.052 (.033)	−.238 (.057)	−.102 (.052)
Negative Display Rules	.109* (.052)	.080** (.031)	.083** (.019)
Surface Acting	.260*** (.067)	.288* (.111)	0.220*** (.032)
Deep Acting	.049 (.025)	.049 (.025)	−.049 (.085)
*Respondent Demographics*			
Gender			
Female	.216 (.206)	.224 (.206)	.221 (.206)
Other	1.417 (.729)	1.435* (.728)	1.403 (.726)
Age	−.026*** (.004)	−.026*** (.007)	−.027*** (.007)
White^a^	.189 (.182)	.189 (.182)	.187 (.183)
*Work Context*			
Autonomy	−.181 (.095)	−.183 (.095)	−.179 (.095)
Site			
Libraries^b^	−.698*** (.179)	−.699*** (.179)	−.697*** (.179)
Authority	.087 (.270)	.087 (.270)	.091 (.270)
Client contact	.163 (.190)	.163 (.190)	.180 (.190)
*Interactions*			
Negative Display Rules × Surface Acting	−.005 (.008)	–	–
Positive Display Rules × Surface Acting	–	−.005 (.009)	–
Positive Display Rules × Deep Acting	–	–	−.008 (.007)
Constant	−.699 (.663)	−.871 (.822)	.076 (.779)
Pseudo *R*^2^	.138	.138	.139

*Note*. *R*^2^ represents the mean across 10 imputations. Standard errors included in parentheses.

a vs other.

b vs mental health professionals.

* *p* < .05. ***p* < .01. ****p* < .001.
